# Supplementary material for: Distribution and characteristics of androgen receptor (AR) in breast cancer among women in Addis Ababa, Ethiopia: A cross sectional study
Source: PLoS One. 2020 May 6;15(5):e0232519. doi: 10.1371/journal.pone.0232519 (PMC7202607; doi:10.1371/journal.pone.0232519)
Supplement: Attachment — Submitted filename: Response to Reviewers.docx [file pone.0232519.s002.docx]

**Responses to Reviewers**

**Title:** Distribution and characteristics of androgen receptor (AR) in breast cancer among women in Addis Ababa, Ethiopia: A cross sectional study

**Authors:**

Endale Hadgu ([endalehadgu@yahoo.com](mailto:endalehadgu@yahoo.com))

Daniel Seifu (daniel.seifu@aau.edu)

Wondemagegnhu Tigneh ([tigeneh@yahoo.com](mailto:tigeneh@yahoo.com))

Yonas Bokretsion ([yonasbtsion@yahoo.com](mailto:yonasbtsion@yahoo.com))

Abebe Bekele (abebesurg@yahoo.com)

Markos Abebe ([markosabebe@yahoo.com](mailto:markosabebe@yahoo.com))

Thomas Sollie (tomas.sollie@regionorebrolan.se)

Christina Karlsson ([christina.karlsson@oru.se](mailto:christina.karlsson@oru.se))

Mats G Karlsson ([mats.g.karlsson@regionorebrolan.se](mailto:mats.g.karlsson@regionorebrolan.se))

Dear Editors,

We would like to thank you for giving us the opportunity to re-submit our manuscript to PLOS ONE. Thank you very much for reviewing the paper and giving valuable feedback. We have addressed the academic editor and reviewers’ comments in the resubmitted draft, with changes highlighted. We would like to thank the academic editor and reviewers for their constructive ideas which have helped improve comprehension for the audience.

We provide here a letter with a point-by-point response to the concerns:

**Response to the academic editor:**

We thank the academic editor for the valuable comments!

Please find our responses:

1. Please ensure that your manuscript meets PLOS ONE's style requirements, including those for file naming.

>>>This is addressed in the revision

2. We noticed minor instances of text overlap with the following previous publication(s), which need to be addressed:

<https://bmcwomenshealth.biomedcentral.com/articles/10.1186/s12905-018-0531-2>

 The text that needs to be addressed involves the Abstract and the Methods section.

 In your revision please ensure you cite all your sources (including your own works), and quote or rephrase any duplicated text.

>>>This is addressed in the revision

3. Please provide additional details regarding participant consent. In the Methods section, please ensure that you have specified (1) whether consent was informed and (2) what type you obtained (for instance, written or verbal). If your study included minors, state whether you obtained consent from parents or guardians. If the need for consent was waived by the ethics committee, please include this information.

>>>This is addressed in the revision. Separate Ethics statement part is included in the methods part. No minors were included in our study

4. In the ethics statement in the manuscript and in the online submission form, please provide additional information about the patient records used in your retrospective study, including: a) whether all tissue samples were fully anonymized before you accessed them; b) the date range (month and year) during which patients' medical records were accessed; c) the source of the medical records analyzed in this work (e.g. hospital, institution or medical center name). If patients provided informed written consent to have data from their medical records used in research, please include this information.

>>>This is addressed in the revision.

5. Please amend either the title on the online submission form (via Edit Submission) or the title in the manuscript so that they are identical.

>>>This is addressed in the revision

6. Please upload a copy of Figure 1, to which you refer in your text on page 9. If the figure is no longer to be included as part of the submission, please remove all reference to it within the text.

>>>Figure 1 was already uploaded with our submission under attach files as Figure. Our figure is submitted in a file name S1 and labeled as **Figure 1** Representative images of AR positive immunohistochemistry in breast tumor tissue array. **A,** H&E staining (10X); **B**, immunostaining (10X); **C,** immunostaining of a specific area (40X). The file name is now corrected to S1 Fig and uploaded.

**Reviewer 1**

We thank the reviewer for the valuable comments!

Please find our responses:

1. While most clinical trial report the use of specific antibody (AR 441 from DAKO) to address the antibody related controversy over variable AR expression, the authors’ do not provide any rationale for using a different antibody for staining AR. Moreover, looking at micrographs showing AR staining in Figure 1, it seems that there is a huge amount of background/non-specific staining and scoring these type of staining may have a false positive results. The authors might have to provide micrographs for positive and negative controls used.

>>> We don’t have any special reason to use the Anti androgen receptor antibody clone EP 120 from Epitomics. It was the clone available in the laboratory at the time. We have supplied the micrograph for the positive and negative controls used in the study.

2. Multiple factors like sample procuring method, antibody used to stain and method of staining and scoring (different cut off values) dictate the discrepancy in the results of expression of AR in TNBC. The paper do not address any of these issues.

>>> The comment is correct, and we have addressed the issue under our discussion part in paragraph 4 page 12.

3. There is a recent paper from Bhattarai [et.al](http://et.al/) on prognostic role of AR in global TNBC cohort where the authors talk about AR expression in various cohorts including African cohort. It would be worth including the paper in the discussion.

>>>We have included the paper in our discussion.

4. Since Breast cancer patients of African ancestry have aggressive TNBC phenotype, it would be worth looking at the survival trend between AR+ and AR- TNBCs.

>>>This is a very good recommendation and would consider comparing survival in other study.

5. In addition, the manuscript has instances of awkward English and stylistic concerns so it would be great if the authors pay a little attention on spelling and sentence constructions.

>>>We think this is addressed in the revision

**Reviewer 2**

We thank the reviewer for valuable comments!

Please find our responses:

1. Table 3 shows that there are no significant differences in clinico-pathological parameters between tumors that express AR and those that do not. If this is the case, what does this say about the role that AR plays in tumor behavior in Ethiopian BCa? How will therapies that target AR make a difference? It seems that this data supports that AR is a marker that is present in the majority of Ethiopian BCa, but it does not say anything about its prognostic usefulness. Given the number of samples and the percentage of samples that are AR positive, the authors have already agreed that the study would be stronger with the increase in number of samples. It is possible that the low number of AR negative samples are masked in the measure of differences in clinico-pathological parameters.

   Therefore, while I agree that the study shows, as others have shown, that AR expression is high in ER positive BCa, I do not agree that THIS study shows that Ethiopian BCa could benefit from AR targeted treatments. It would be helpful if the study could show survival or treatment data that would allow a clearer picture of the prognostic value of AR in this population. Increasing the numbers of samples may indeed show significant differences in clinical parameters.

>>> This is correct. Previous studies assessing the role of AR in breast cancer showed its positive prognostic role in hormone receptor positive BCa and its therapeutic significance in TNBC and is presented in our paper. The lack of association between AR expression and clinicaopathological parameters in our study is highly likely due to small sample size and is stated in the discussion part of the paper. We agree that survival or treatment data would give a clear picture and hope future studies to address these issues.

2. The submitted manuscript needs significant editing regarding grammar and clarity. The first part of the discussion is almost a word-for-word repeat of the introduction instead of a summary of the study and overall findings. Also, there is no need to include a statement saying that you aren't going to re-hash the methods portion of the previous study. Either briefly describe the methods from the previous study with explanation of AR specific portions of these methods and reference to your previous study, or re-write the methods. It seems that you have re-written the methods anyway.

>>> We agree with your concern and have edited the paper in the resubmission.

3. Please do not use the word "about" when you are referring to the number of samples. Give the absolute number and describe in detail what it means for a sample to be "evaluable."

>>> We agree with your concern and removed the word “about” and have added more information to explain what evaluable is.

With kind regards!

Endale Hadgu
