# Supplementary material for: Distribution and characteristics of androgen receptor (AR) in breast cancer among women in Addis Ababa, Ethiopia: A cross sectional study
Source: PLoS One. 2020 May 6;15(5):e0232519. doi: 10.1371/journal.pone.0232519 (PMC7202607; doi:10.1371/journal.pone.0232519)
Supplement: Attachment — Submitted filename: Response to Reviewers.docx [file pone.0232519.s003.docx]

**Responses to Reviewers**

**Title:** Distribution and characteristics of androgen receptor (AR) in breast cancer among women in Addis Ababa, Ethiopia: A cross sectional study

**Authors:**

Endale Hadgu ([endalehadgu@yahoo.com](mailto:endalehadgu@yahoo.com))

Daniel Seifu (daniel.seifu@aau.edu)

Wondemagegnhu Tigneh ([tigeneh@yahoo.com](mailto:tigeneh@yahoo.com))

Yonas Bokretsion ([yonasbtsion@yahoo.com](mailto:yonasbtsion@yahoo.com))

Abebe Bekele (abebesurg@yahoo.com)

Markos Abebe ([markosabebe@yahoo.com](mailto:markosabebe@yahoo.com))

Thomas Sollie (tomas.sollie@regionorebrolan.se)

Christina Karlsson ([christina.karlsson@oru.se](mailto:christina.karlsson@oru.se))

Mats G Karlsson ([mats.g.karlsson@regionorebrolan.se](mailto:mats.g.karlsson@regionorebrolan.se))

Dear Editors,

We would like to thank you again for giving us the opportunity to re-submit our manuscript to PLOS ONE. Thank you very much for reviewing the paper and giving valuable feedback. We have addressed the academic editor and reviewers’ comments in the resubmitted draft, with changes highlighted. We would like to thank the academic editor and reviewers for their constructive ideas which have helped improve comprehension for the audience.

We provide here a letter with a point-by-point response to the concerns:

**Response to the reviewers:**

**Reviewer 1**

We thank the reviewer for the valuable comments!

Please find our responses:

1. The manuscript still has instances of awkward english and foremost this is not a novel study as there are studies assessing the expression of AR in African women. The sample size is very low and unless the survival analysis is performed we cannot mark the importance of this study. It would be impactful if the authors increase the sample size and see if there is any association of AR expression with clinicopathological parameters and perform the survival analysis to evaluate its prognostic significance.

>>> We have made an extensive language editing in this revision and hope to answer your question.

>>> Concerning sample size, we acknowledge an increased sample size would provide a better insight into the study. We clearly indicated this as a one of the limitations and hope would be addressed by future studies.

>>>We agree survival study could reveal a better information, but we don’t have data to do survival analysis.

**Reviewer 3**

We thank the reviewer for valuable comments!

Please find our responses:

1. Skewed continuous variables (age) should be tested for differences using a non-parametric test, such as the Wilcoxon rank-sum test.

>>> This is correct and addressed in the revision.

2. The Materials and Methods section repeats text verbatim from earlier published work. Paraphrasing is needed. Additionally, the citation to the earlier work can be referenced once at the beginning of the Methods section and noted that this paper briefly describes those same methods.

>>> We agree with your concern and have edited the paper in the revision.

3. The largest issue to address is in the Discussion when relating the present study to the literature. While differences may be due to true differences across sub-Saharan African populations, there are other possible explanations that should be addressed. This includes the use of an antibody in this study that may result in an overestimate of AR prevalence compared to other work with AR 441. Could this be why the prevalence of AR-positive BCa is higher in this study compared to others? A second factor, as the authors point out, is the cutoff score of 1% versus 10%. Is it possible with the present data to provide AR-positive prevalence using the 10% cutoff to allow for a direct comparison? Would how the participants are enrolled or the samples collected impact findings across the studies?

>>> We agree with you concerning the antibody clone and have included your indication in the revision.

>>> AR is currently interpreted at a cutoff of 1% and it is our hope that more studies would be conducted on AR in this continent and a future comparative study would possibly come up with a better insight.

With kind regards!

Endale Hadgu
